# Supplementary figures and images for: Targeted NGS for species level phylogenomics: “made to measure” or “one size fits all”?
Source: PeerJ. 2017 Jul 25;5:e3569. doi: 10.7717/peerj.3569 (PMC5530999; doi:10.7717/peerj.3569)

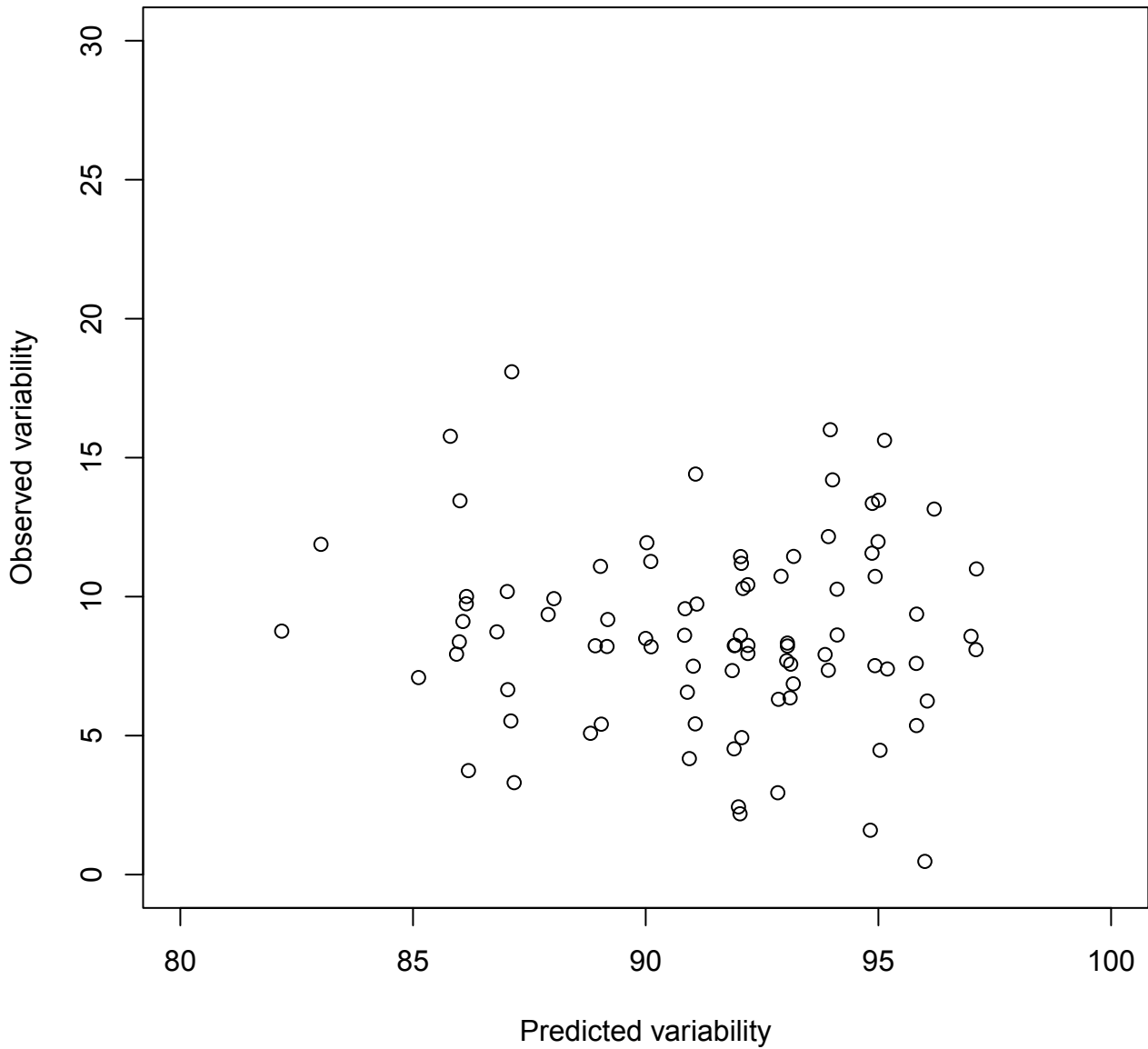

Supplement: Data S5 — Plot of sequence similarity (transcriptome data; Rhododendron and Vaccinium) against sequence similarity (empirical dataset generated here; Rhododendron and Erica spp.) for individual markers. [file peerj-05-3569-s005.pdf]

**A**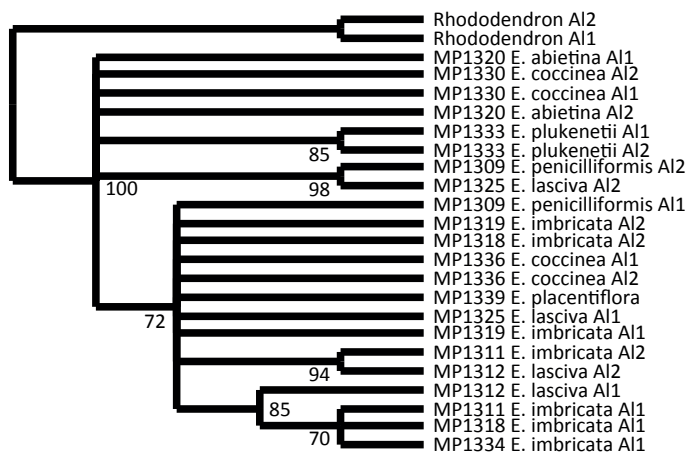**B**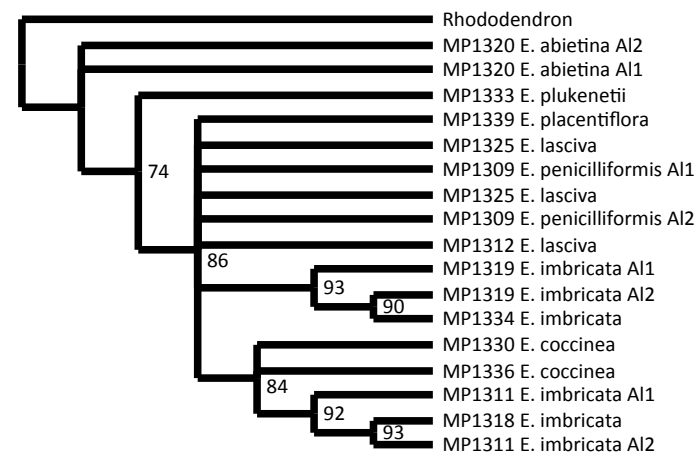**C**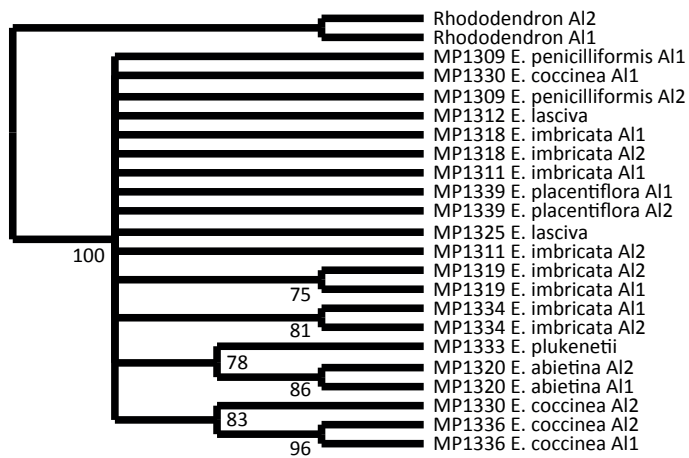**D**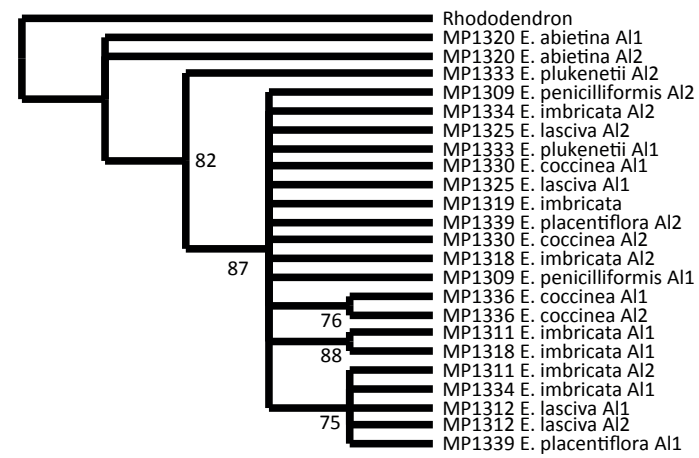**E**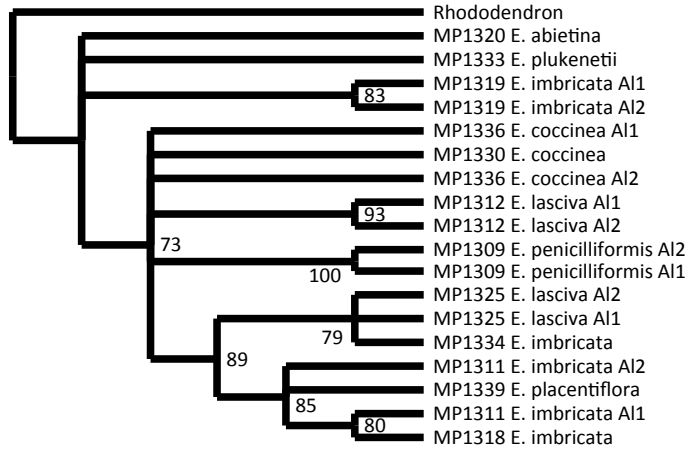**F**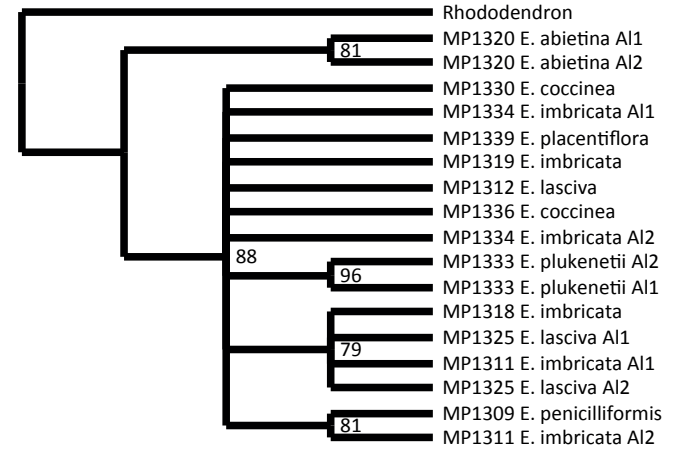**G**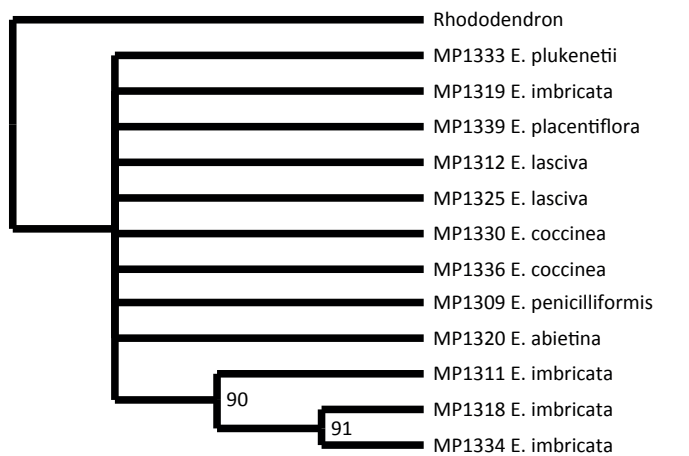**H**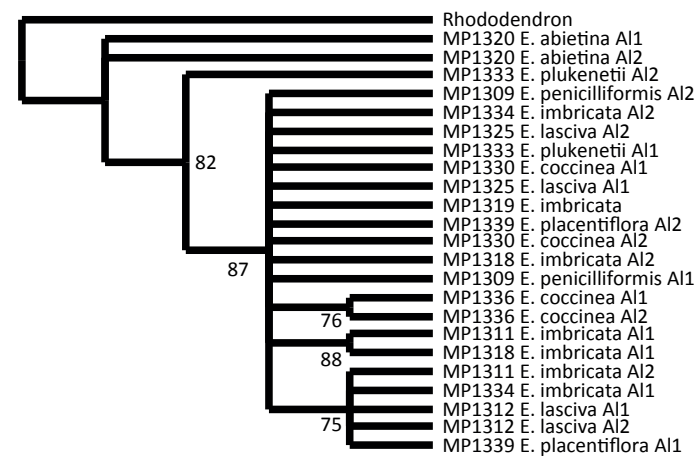

Supplement: Figure S1 — The six nuclear markers that delivered the greatest numbers of nodes supported by ≥70% BS (A: marker 4430; B: 12303; C: 14220; D: 17845; E: 20893; F: 22868) are presented along with those based on mitochondrial (G) and ITS (F) sequences. Terminals correspond to collection codes and species names (Table 1). Some taxa are represented twice in some trees due to the presence of alleles, including two distinct copies of ITS in E. abietina ssp. aurantiaca (confirming previous work using cloning; Pirie et al., in press). Node labels represent bootstrap support. [file peerj-05-3569-s006.pdf]
